# Potential crosstalk between SPP1 + TAMs and CD8 + exhausted T cells promotes an immunosuppressive environment in gastric metastatic cancer

**DOI:** 10.1186/s12967-023-04688-1

**Published:** 2024-02-16

**Authors:** Yan Du, Yilin Lin, Lin Gan, Shuo Wang, Shuang Chen, Chen Li, Sen Hou, Bozhi Hu, Bo Wang, Yingjiang Ye, Zhanlong Shen

**Affiliations:** 1 Department of Gastroenterological Surgery, Peking University People’s Hospital, Beijing, China; 2 Laboratory of Surgical Oncology, Peking University People’s Hospital, Beijing, China; 3 Beijing Key Laboratory of Colorectal Cancer Diagnosis and Treatment Research, Beijing, China

**Keywords:** Gastric metastatic cancer, SPP1 + TAMs, CD8 + T exhausted cells, Immunosuppressive, Single-cell analysis

## Abstract

**Background:**

Immunotherapy brings new hope to patients with advanced gastric cancer. However, liver metastases can reduce the efficacy of immunotherapy in patients. Tumor-associated macrophages (TAMs) may be the cause of this reduction in efficacy. SPP1 + TAMs are considered to have immunosuppressive properties. We aimed to investigate the involvement of SPP1 + TAMs in the metastasis of gastric cancer.

**Methods:**

The single-cell transcriptome was combined with batched BULK datasets for analysis. Animal models were used to verify the analysis results.

**Results:**

We reveal the interaction of SPP1 + TAMs with CD8 + exhausted T cells in metastatic cancer. Among these interactions, GDF15-TGFBR2 may play a key immunosuppressive role. We constructed an LR score to quantify interactions based on ligands and receptors. The LR score is highly correlated with various immune features and clinical molecular subtypes. The LR score may also guide the prediction of the efficacy of immunotherapy and prognosis.

**Conclusions:**

The crosstalk between SPP1 + TAMs and CD8 + exhausted T cells plays a key immunosuppressive role in the gastric metastatic cancer microenvironment.

**Graphical Abstract:**

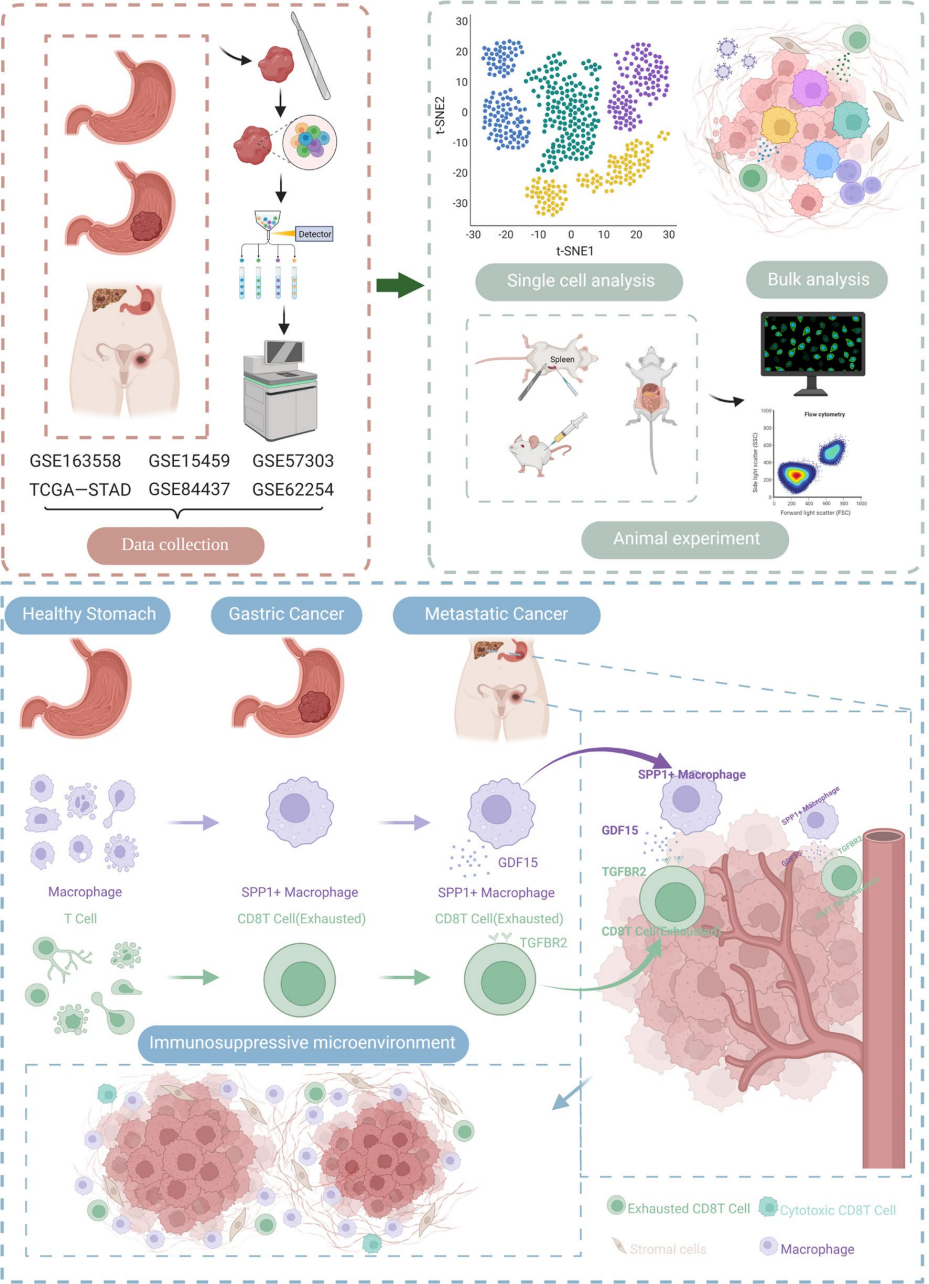

**Supplementary Information:**

The online version contains supplementary material available at 10.1186/s12967-023-04688-1.

## Introduction

Gastric cancer poses a significant threat to the well-being of the worldwide populace. Its morbidity and mortality rank among the top five of all malignant tumors [1–3]. Gastric cancer has the second highest incidence and death rate among malignant tumors in China. The death rate of gastric cancer in China is significantly greater than that of the majority of developed nations [4]. The situation in China is concerning. Some progress has been made in the etiology, diagnosis and treatment of gastric cancer in recent years. However, the prognosis for these patients remains poor. Sixty percent of patients still have recurrence and liver metastasis after treatment [5, 6]. Patients diagnosed with advanced gastric cancer typically have a median overall survival rate of merely 1 year, as reported in studies [7, 8]. Therefore, exploring new mechanisms affecting liver metastasis of gastric cancer is an important strategy for improving the prognosis of gastric cancer patients.

In recent years, it has been discovered that the tumor microenvironment is widely involved in the mechanism of tumor metastasis. Tumor-infiltrating CD8 + T cells recognize and kill tumor cells and are soldiers of immunotherapy [9, 10]. However, tumor cells may inhibit the killing ability of CD8 + T cells through the immune checkpoint (PD-1/PD-L1) signaling pathway, thereby inducing immune escape. Therefore, the emergence of immune checkpoint blockade (ICB) drugs based on tumor microenvironment characteristics has brought new benefits to patients with advanced gastric cancer [11]. The REGONIVO trial and ATTRACTION-2 trial show the efficacy of PD-1 drugs plus targeted therapy in chemotherapy-refractory patients [12, 13]. The ORIENT-16 and CheckMate-649 trials demonstrated that the combination of PD-1 drugs and chemotherapy is an effective initial treatment option for patients who are not eligible for surgery [14]. The KEYNOTE-811 study demonstrated that the combination of PD-1 drugs, targeted therapy, and chemotherapy can greatly enhance the objective response rate in individuals with HER2-positive gastric cancer [15]. Nevertheless, only a limited proportion of individuals diagnosed with gastric cancer can reap benefits of immunotherapy. In some cases, immunotherapy may even accelerate tumor progression [16]. This is mainly due to the high heterogeneity of the tumor and its surrounding complex ecosystem [17]. Through a wide range of cancer and noncancer cell interactions. Cancer cells can form an active immunosuppressive microenvironment [18]. Hence, high-resolution characterization of the microenvironment of tumor remodeling and interactions between cells is of great significance for revealing the characteristics of the tumor microenvironment that cause metastasis in gastric cancer patients and improving the efficacy of immunotherapy.

Furthermore, several research studies have indicated that liver metastasis can greatly diminish the effectiveness of immunotherapy in individuals [19–21]. Nevertheless, studies conducted on animals before clinical trials have discovered that liver metastases can siphon activated CD8 + T cells into the systemic circulation. This siphoning function leads to the emergence of ‘‘immune deserts’’ [19]. More tumor-associated macrophages (TAMs) are found in liver metastases and may be the cause [19]. TAMs may inhibit the killing ability of CD8 + T cells by secreting a variety of cytokines, leading to immune suppression. There are extensive research reports suggesting that macrophages exist in an M1/M2 dual polarization state in vitro [22]. However, M1 and M2 signature genes can be coexpressed in almost all TAMs [23]. Furthermore, M1 and M2 features in TAMs are not mutually exclusive [24]. Therefore, the intrinsic mechanism may not be resolved based on traditional macrophage classification. The development of single-cell transcriptome sequencing provides favorable conditions for comprehensively revealing the characteristics of cell subpopulations in the tumor microenvironment. It was recently reported that based on single-cell transcriptome sequencing, TAMs contain a uniquely characterized subpopulation called SPP1 + TAMs. This subpopulation has immunosuppressive properties [25]. The study found that the immunosuppressive microenvironment of gastric cancer is dynamically related to the emergence of SPP1 + TAMs during anti-PD-1 immunotherapy [17]. Other studies have found that SPP1 + TAMs can interact with tumor-associated fibroblasts and prevent lymphocytes from infiltrating the tumor core [26]. Nonetheless, the involvement of SPP1 + TAMs in the metastasis of gastric cancer remains unclear.

During our investigation, we collected single-cell transcriptome data from healthy stomachs, primary gastric cancers, and gastric metastases. Five common gastric cancer BULK datasets were integrated. Our study contains the National Cancer Institute (TCGA-STAD) and Asian Cancer Research Group (ACRG, GSE62254) datasets. We first characterized CD8 + T cells and macrophages in different disease states. The interactions between SPP1 + TAM cells and CD8 + exhausted T cells was significantly enhanced in metastasis. Among these interactions, GDF15-TGFBR2 may play a key immunosuppressive role. It has the potential to improve the expression of coinhibitory receptors in exhausted CD8 + T cells, leading to eventual apoptosis. This effect was verified by immunofluorescence and flow cytometry. Moreover, a score was created to measure the interaction between SPP1 + TAMs and CD8 + exhausted T cells. The interaction score is highly correlated with various immune characteristics and clinical molecular subtypes. The interaction score may guide the prediction of the efficacy of immunotherapy and prognosis.

## Methods

### Data retrieval and sources

The GEO database (GSE163558) [27] provided single-cell transcriptome data. Multiple sets of BULK transcriptome data were derived from TCGA-STAD, GSE15459, GSE57303, GSE62254 (ACRG), and GSE84437. TCGA-STAD data convert expression data to FPKM. The BULK dataset was normalized using the ‘‘AFFY’’ and ‘‘SIMPLEAFFY’’ R packages. The BULK dataset underwent batch correction using the R package ‘‘SVA’’. The clinical data were acquired from the corresponding cohorts.

### Processing of single-cell data

The ‘‘SEURAT’’ R package [28] was utilized to process and visualize the single-cell data. First, the samples with a COUNT number greater than 100,000, a gene expression number less than 200 or greater than 8000, and a mitochondrial gene proportion greater than 20% were filtered (Additional file 1: Fig. S1A). Next, the data underwent normalization using the ‘‘NORMALIZEDATA’’ function and the ‘‘SCALEDAT’’ function. Data were found for hypervariable genes using the ‘‘FINDVARIABLEFEATURES’’ function. The data were subjected to principal component analysis based on hypervariable genes to determine the dimensionality reduction. Finally, clustering, projection (UMAP) and annotation of cell clusters are performed.

### Gene signature score

The ‘‘ADDMODULESCORE’’ function in the ‘‘SEURAT’’ R package was used to calculate the ‘‘Gastric Cancer’’ score, ‘‘Epithelial Mesenchymal Transition’’ score, ‘‘Cell Motility’’ score, ‘‘Cell Cycle’’ score, ‘‘M1’’ score, ‘‘M2’’ score, ‘‘Phagocytosis’’ score, and ‘‘Angiogenesis’’ score on single-cell data [23, 29] (Additional file 2: Tables S1, S2, S3). The ‘‘ssGSEA’’ function in the ‘‘GSVA’’ R package was used to calculate the ‘‘GDF15 + SPP1 + TAM’’ score, ‘‘CD8 + T exhausted cell’’ score, ‘‘PDCD1 + CD8 + T-cell’’ score, ‘‘TIGIT + CD8 + T-cell’’ score, ‘‘CTLA4 + CD8 + T’’ score, ‘‘HAVCR2 + CD8 + T-cell’’ score, ‘‘LAG3 + CD8 + T-cell’’ and ‘‘CXCL13 + CD8 + T-cell’’ on BULK data (Additional file 2: Table S4).

### Trajectory analysis

We assessed the developmental trajectories of different subsets of CD8 + T cells [30] by using the ‘‘MONOCLE’’ R package.

### Analysis of cell‒cell interactions

We used the ‘‘CELLPHONEDB’’ [31] and ‘‘CELLCHAT’’ [32] R packages for high-resolution characterization of cell‒cell interactions. According to the official workflow. The ‘‘CELLPHONEDB’’ R package calculates the potential interaction strength between cells. The ‘‘CELLCHA’’ R package calculates the contribution of different cells to the interaction strength. It visualizes detailed signaling pathways, interaction patterns and each ligand‒receptor pair [32].

### Cell culture

MCF cells were purchased from (Procell, China). MFC cells were cultured in RPMI 1640 medium (Gibco, USA) supplemented with 10% fetal bovine serum (Gibco, USA) at 37 °C and a CO_2_ concentration of 5%.

### Mouse liver metastasis model

In this study, the animal of origin of MFC cells was used: mouse (615 Mouse). The mouse (615 Mouse) was purchased from (Wukong Biotechnology, China). Eight mice (615 Mouse) were fed and managed according to SPF level. When the MFC cells were grown in an incubator to 80% confluence, the cells were digested using pancreatic enzymes (Gibco, USA) and finally resuspended in phosphate-buffered saline (PBS). Six-week-old 8 mice (615 Mouse) were anesthetized with 1.5% pentobarbital sodium, disinfected with 75% alcohol, and then the left abdominal cavity of the mice was cut open with sterile surgical scissors. A total of 5 × 10^5^ cells (50 µl) were injected under the spleen capsule. A 75% alcohol cotton ball was used to gently press the injection site. The abdomen and the incision were closed with surgical sutures. During recovery from anesthesia, mice were placed under a warming lamp. Mice were monitored weekly by in vitro live imaging. Before monitoring, 200 µl of D-fluorescein potassium salt (MEILUN Cell, China) was injected intraperitoneally, and after 10 min, the sample was placed in an imager (IVIS Spectrum, USA) to observe liver metastasis. Following a three-week period of tumor development, 8 mice (615 Mouse) were euthanized, and liver metastases were obtained. All animal experimental protocols were approved by the Ethics Committee of Peking University People’s Hospital.

### GDF15 inhibitor therapy

The mouse gastric cancer liver metastasis model was prepared as described above. There were 4 mice in the control group and 4 mice in the experimental group. On the seventh day after tumor growth, a GDF15 inhibitor (HY-P99241, MCE) was injected intraperitoneally for treatment (10 mg/kg) in the experimental group, and the same volume of PBS was used for the control group. Afterward, injections were administered every 72 h, resulting in a total of 6 shots. Mice were monitored weekly by in vitro live imaging. Before monitoring, 200 µl of D-fluorescein potassium salt was injected intraperitoneally, and after 10 min, the sample was placed in an imager to observe liver metastasis. After the final treatment, the mice were euthanized on the following day, and liver metastases were gathered. Finally, the number of metastases in each liver was counted. Student’s t test was used for statistical analysis, and P < 0.05 was considered significant.

### H&E staining

Paraffin-embedded liver metastases were sectioned into 3 µm slices and then placed on glass slides. The slides were baked in a 60 °C oven for one hour. The sections were dewaxed, dehydrated, and stained with aqueous hematoxylin and alcohol. After dehydration with absolute alcohol, the sections were cleared with xylene. Gum was dripped onto the transparent sections, and then the slices were placed under a scanner for panoramic imaging (PANORAMIC MIDI, 3DHISTECH, Hungary).

### Multiplex immunofluorescence staining

Paraffin sections of liver metastases were dewaxed, and the tissue sections were then placed in EDTA-filled antigen repair buffer (Servicebio, China) for antigen repair in a microwave oven. After antigen repair was completed, a circle was drawn, and sealing was continued with 3% BSA after hydrogen peroxide sealing. Next, the sealing liquid was shaken off, the prepared primary antibody was added to the slices, and the slices were incubated at 4 °C overnight. After washing, TSA dye was added in drops (Servicebio, China) and incubated for 10 min at room temperature in the dark. Then, the tissue sections were placed in the repair solution and heated in a microwave oven for approximately 10 min to remove the primary and secondary antibodies that had bound to the tissue. The above steps were repeated to add the second, third primary antibody and second antibody, and staining was continued with TSA dye. After antibody staining, DAPI was added to the cell nucleus. Finally, the slices were sealed with a self-fluorescence quencher and placed under a slide digital scanner for panoramic imaging (PANORAMIC MIDI, 3DHISTECH, Hungary). Information about the primary antibodies included CD8 + α (29,896-1-AP, Proteintech), Spp1 (YT3467, Immunoway), Cd68 (28,058-1-AP, Proteintech), and Gdf15 (27,455–1-AP, Proteintech).

### Flow cytometry

The liver metastases were gathered and then fragmented into small fragments measuring 1–2 mm3 using eye scissors. The tissue was treated with a protease solution containing collagenase D (1 mg/ml, CAS-COLLD-RO) and DNase I (0.1 mg/ml, CAS-10104159001) from Merck. The tissue was then incubated at 37 °C for 30 min to facilitate digestion. Following digestion, the cells underwent filtration using a cell strainer with a pore size of 70 μm. The cells were washed once with dye buffer. Staining was performed on cells using anti-CD45 (103138, Biolegend) and anti-CD8 + α (100712, Biolegend). Flow cytometry (CytoFLEX, USA) was used to determine the percentage of CD8 + T cells. Student’s t test was used for statistical analysis, and P < 0.05 was considered significant.

### Interaction-based scoring model building and grouping

We constructed an LR score to quantify interactions based on ligands and receptors. CELLCHAT recognizes all ligands and receptors between the two groups of cells. All ligands and receptors were subjected to Cox regression analysis. The LR score was constructed using the LASSO method. The LR score formula: LR score = ∑_i_ Expression (LR)_i_ * coef_i_.

### Cell infiltration type and correlation analysis

Immune cell infiltration and immune function activity were scored using CIBERSORT and ssGSEA. Spearman was used to assess the correlation with LR scores. You can download TIDE [33] from http://tide.dfci.harvard.edu/.

### Illustration production

All illustrations were created with BioRender.com.

### Statistical analysis

Statistical analyses were performed using R (version 4.1.2), Python (version 3.10) and GraphPad Prism (version 9.2.0). Nonnormally distributed data were analyzed using the Wilcoxon test. Three or more sets of data were analyzed using the Kruskal‒Wallis test. The number of liver metastases and flow analysis were performed by Student’s t test, and P < 0.05 was considered statistically significant.

## Results

### Heterogeneous single-cell landscape in healthy stomach\gastric cancer\metastasis

**Fig. 1:**
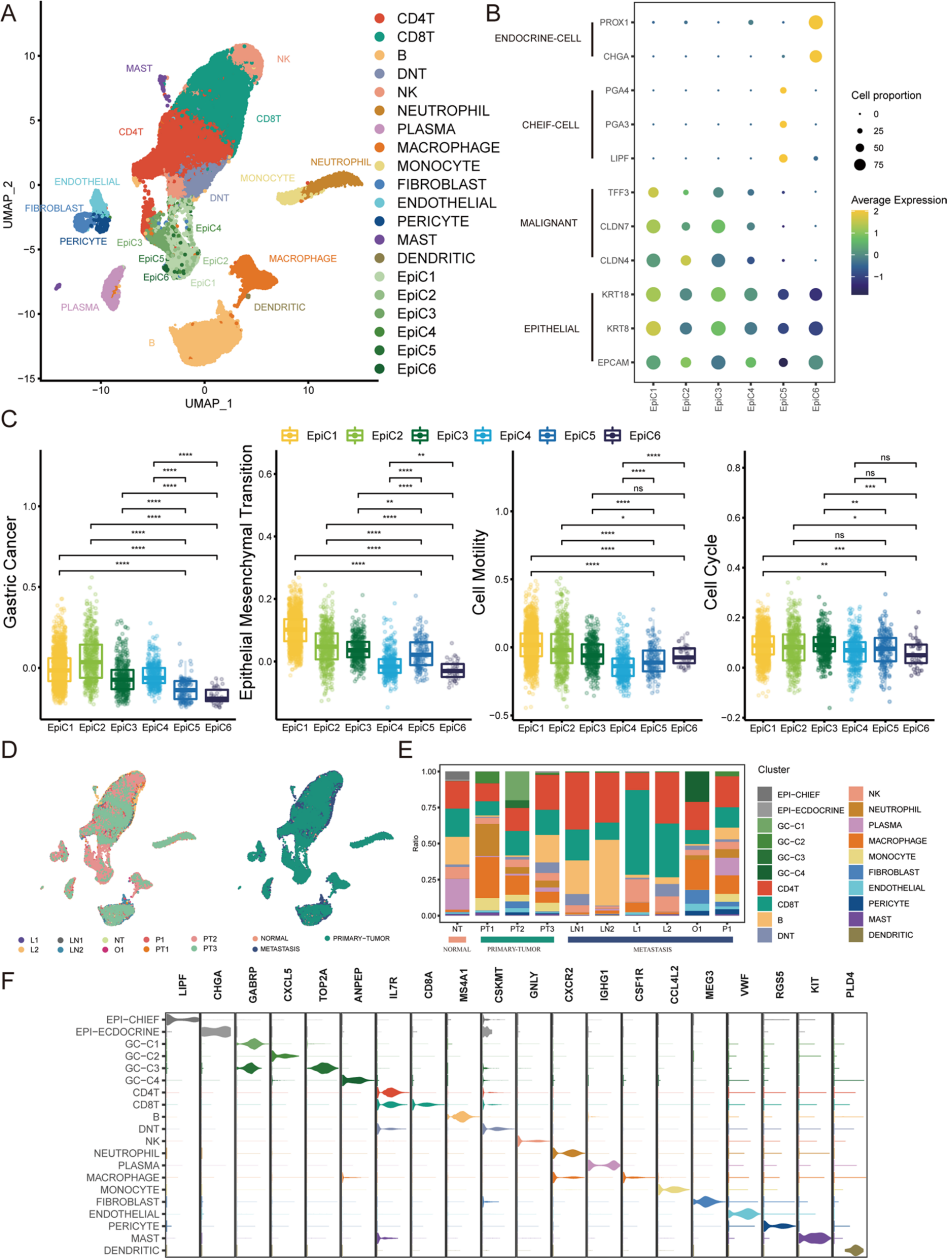
Single-cell landscape of healthy stomach, gastric primary and metastatic cancers. **A**. UMAP diagram showing the 20 major cell types**B** . Bubble heatmap showing the expression of benign/malignant epithelium-specific markers in six epithelial cell subpopulations. CHIEF cells and endocrine cells are normal gastric epithelial cells. **C**. Boxplot showing benign/malignant epithelium-specific functional scores in six epithelial cell subpopulations. **D**. UMAP diagram colored by different disease states. PT1, PT2, and PT3 are derived from gastric primary tumors. NT1 is derived from adjacent nontumors. LN1 and LN2 are derived from lymph node metastases. L1 and L2 are derived from liver metastases. O1 is derived from metastases. P1 is derived from peritoneal metastases. **E**. Scale diagram showing cellular composition in different disease states. EpiC1-C4 was named GC-C1-C4. EpiC5 is named EPI-CHIEF. EpiC6 is named EPI-ECDOCRINE.** F**. Violin plot showing the expression of marker genes for each cell type. Wilcoxon test: *P < 0.05; **P < 0.01; ***P < 0.001; ****P < 0.0001

To characterize the heterogeneous single-cell landscape of healthy stomachs, gastric primary cancers, and metastatic cancers at high resolution, we used a previously published gastric cancer single-cell dataset. Including gastric primary tumors (PT1, PT2, PT3), adjacent nontumors (NT1), and gastric metastases: lymph node metastases (LN1, LN2), liver metastases (L1, L2), ovarian metastases (O1) and peritoneal metastases (P1). After quality control and screening (Additional file 1: Fig. S1A), 44,234 cells were obtained. Cell clusters were defined as 20 independent types via data normalization, principal component analysis, clustering, and cell type identification (Fig. 1A, Additional file 1: Fig. S1B). The high expression of KRT8, KRT18, and EPCAM identified epithelial cells EpiC1-C6. High expression of PECAM1 and COL1A2 identified a stromal cell population, which was further subdivided into endothelial cells (CDH5, VWF, and PLVAP), fibroblasts (FGF7, DCN, and LUM), and pericytes (RGS5 and NOTCH3). High expression of CD45 was identified as immune cell clusters, further subdivided into neutrophils (CD66b, S100A8, S100A9), mast cells (KIT, TPSAB1, CPA3), dendritic cells (PLD4, LILRA4, FCER1A), monocytes (CD14, CD16), macrophages (CD68, CD163, CSF1R), CD4 + T (CD4), CD8 + T (CD8A, CD8B), DNT (CD2, CD3D, CD4-/CD8-), NK (FGFBP2, CX3CR1, KLRD1), B (CD19, CD79A, MS4A1) and plasma cells (IGHG1, CD79A, TNFRSF17).

Only approximately 25% of malignant gastric epithelial cells exhibit high levels of copy number variation (CNV) [34, 35]. The approach of using CNVs to identify malignant cells was not applicable to gastric cancer. To distinguish between malignant and nonmalignant epithelium, we first used a panel of genes specific for gastric malignant and nonmalignant epithelium. Malignant epithelial genes include TFF3, CLDN7, and CLDN4. These genes were confirmed to be highly expressed in gastric cancer tissues (p < 2 × 10^–16^) [27]. Nonmalignant epithelial genes included chief cell markers (PGA4, PGA3, LIPF) and endocrine cell markers (PROX1, CHGA). These genes are mainly related to gastric digestive enzymes and mucus secretion. Malignant epithelial genes were highly expressed in EpiC1-C4 (Fig. 1B). Endocrine cell markers were highly expressed in EpiC6 cells (Fig. 1B). Chief cell markers were highly expressed in EpiC5 cells (Fig. 1B). This result is further supported by the gene signature set score [29]. EpiC1-C4 showed higher gastric cancer scores (Fig. 1C). Interestingly, EpiC4 exhibited lower EMT and cell motility and a slower cell cycle than EpiC1-C3 (Fig. 1C, Additional file 2: Table S1). We named EpiC1-C4 GC-C1-C4. EpiC5 was named EPI-CHIEF. EpiC6 was named EPI-ECDOCRINE. GC-C1 and GC-C3 were mainly present in the primary tumor (Fig. 1D, E). GC-C4 was mainly present in metastases (Fig. 1D, E). Immune cells accounted for the highest proportion in normal tissue (92.01%), primary tumor (77.37%) and metastatic tumor (95.37%) samples (Fig. 1D, E). The percentage of cancer cells in the primary tumor (16.26%) was significantly greater than that in the metastatic tumor (2.06%) (Fig. 1D, E). Nonetheless, this variation may also be attributed to the relatively small size of metastatic tumors and limited clinical materials. Marker genes of different cell types (Fig. 1F).

**Fig. 2:**
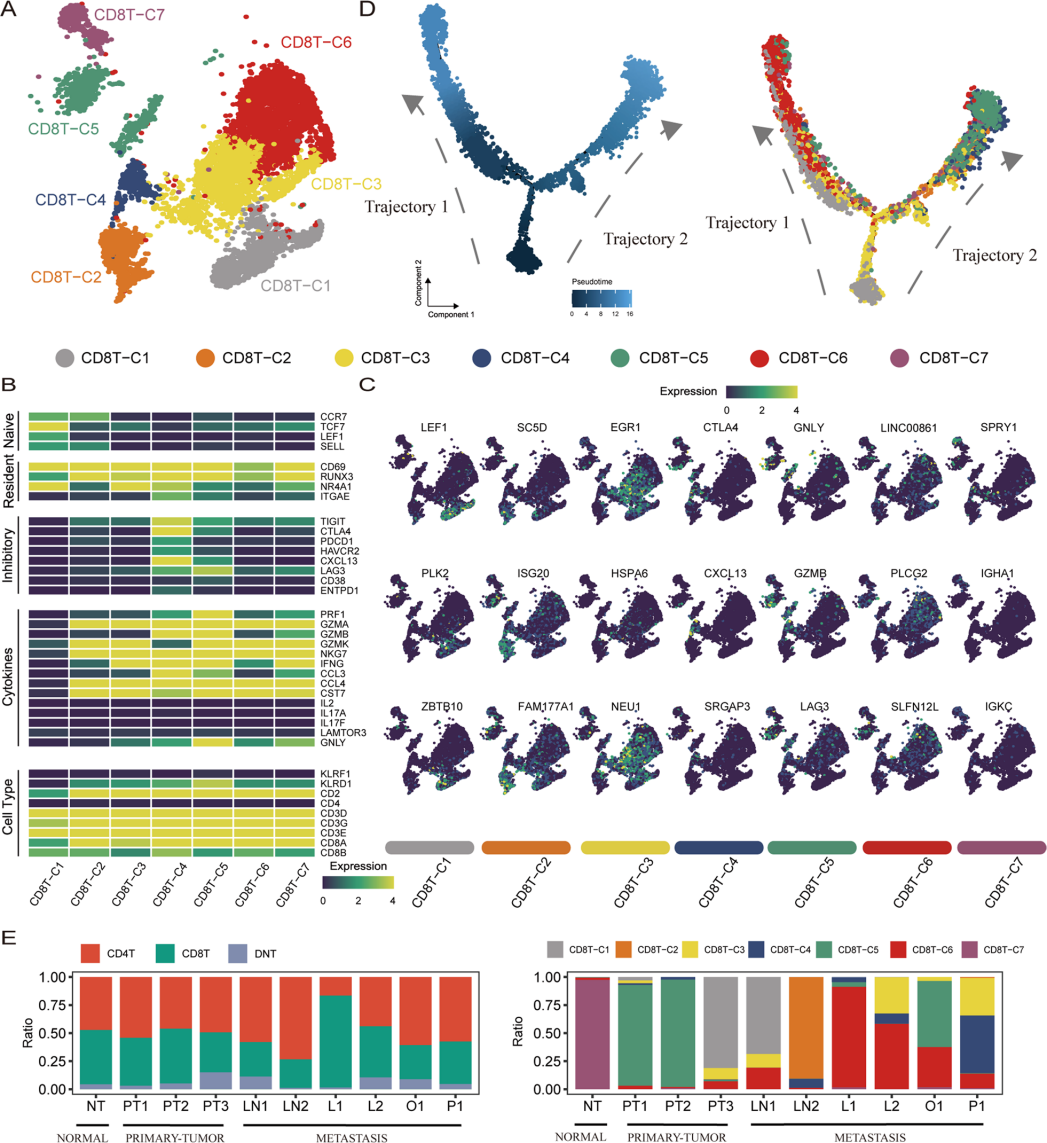
Inferring the cellular state of patient CD8 + T cells. **A**. UMAP diagram showing the 7 major CD8 + T-cell subsets **B**. Heatmap showing the expression of classic CD8 + T-cell function markers for different CD8 + T-cell subsets. CD8T-C4 was defined as an exhausted CD8 + T state. **C**. UMAP diagram showing each CD8 + T-cell subset major type-specific marker. **D**. Trajectory distribution diagram showing the position of each CD8 + T-cell subset in the differentiation process. The color changes from dark to light to simulate the start to end of the pseudotime process. **E**. Scale diagram showing T-cell types and CD8 + T-cell subset cellular composition in different disease states.

The above analysis focuses on the study of tumor cells. However, CD8 + T cells are immune cells that directly kill tumors and play an important role in the process of liver metastasis. Cell clusters were divided into 7 types of CD8TC1-C7 cells by graphical clustering of 9591 CD8 + T cells (Fig. 2A). Classical cell markers indicated the cell status of CD8 + T cells (Fig. 2B). Clusters C1 and C2 enriched for naive-associated genes (CCR7, TCF7, LEF1, SELL) were defined as naive-like CD8 + T status (Fig. 2B). Cluster C3 exhibits resident-associated genes (CD69, RUNX3, NR4A1) defined as the tissue resident memory CD8 + T state (Fig. 2B). Cluster C4, exhibiting inhibitory-associated genes (TIGIT, CTLA4, PDCD1, HAVCR2, CXCL13, LAG3), was defined as an exhausted CD8 + T state (Fig. 2B). Cluster C5 has an intermediate state between the effector CD8 + T state and the exhausted CD8 + T state due to moderate expression of inhibitory-related genes and high expression of cytotoxicity-related genes (Fig. 2B). Clusters C6 and C7, exhibiting cytotoxicity-related genes (GZMA, GZMB, GZMK, NKG7, and IFNG), were defined as effector CD8 + T cells (Fig. 2B). Marker genes of different cell clusters (Fig. 2C). The cells mentioned above, in various states, align with the ongoing progression process in the trajectory analysis (Fig. 2D). The proportion of CD8 + T cells in metastases was the lowest (39.98%), followed by primary tumors (43.75%) and normal gastric tissues (48.40%) (Fig. 2E). The proportion of CD8T-C4 cells in the exhausted state was much higher in metastases (6.48%) than in primary tumors (0.59%) (Fig. 2E).

**Fig. 3:**
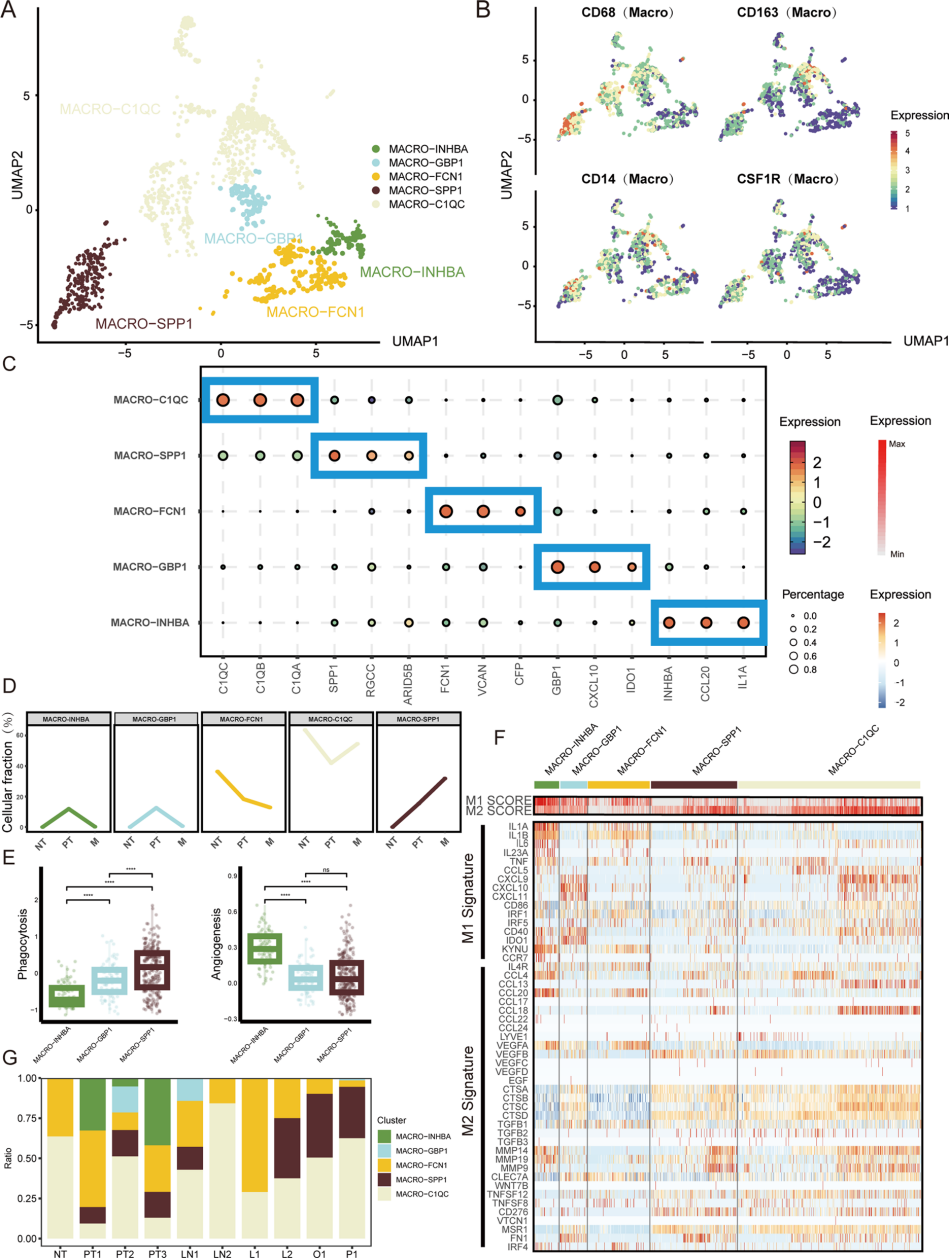
Subclustering and annotation of macrophages. **A**. UMAP diagram showing 5 major macrophage subsets. Based on their type-specific markers, we named them MACRO-C1QC, MACRO-SPP1, MACRO-FCN1, MACRO-GBP1, and MACRO-INHBA. **B**. UMAP diagram showing classic characteristic marker gene expression of 5 major macrophage subsets. **C**. Bubble heatmap showing the expression of major type-specific markers in different macrophage subsets. **D**. Line graph showing disease state preference for different macrophage subsets. MACRO-SPP1, MACRO-INHBA, and MACRO-GBP1 subgroups were not found in normal gastric tissue. These 3 cell types displayed tumor-associated macrophages. MACRO-SPP1 cells were defined as SPP1 + TAMs. **E**. Boxplot showing the functional characteristic scores of different tumor-associated macrophage subsets. **F**. Heatmap showing M1/M2 signature scores and expression of M1/M2 signature genes for different macrophage subsets. **G**. Scale diagram showing different macrophage subset cellular compositions in different disease states. Wilcoxon test: *P < 0.05; **P < 0.01; ***P < 0.001; ****P < 0.0001.

It has been found that tumor-associated macrophages may affect tumor cells and CD8 + T cells. Therefore, a more detailed subgroup of macrophages in this sample was constructed. Cell clusters were classified into 5 types by clustering and identifying 1168 macrophages (Fig. 3A). The classical macrophage genes CD68, CD163, CD14, and CSF1R were all specifically expressed in the five cell subsets (Fig. 3B). Gastric cancer was previously reported to contain two distinct macrophage subsets (C1QC/INHBA) [23]. Other studies have reported that SPP1 + /C1QC + and SPP1 + /C1QC-, SPP1-/C1QC + macrophage subtypes coexist in gastric cancer [36]. Our findings are consistent with these results (Fig. 3C). The five types of macrophage subset marker genes are shown in Fig. 3C. The MACRO-SPP1, MACRO-INHBA, and MACRO-GBP1 subgroups were not found in normal gastric tissue (Fig. 3D). These 3 cell types displayed tumor-associated properties. We assessed the phagocytic and angiogenic capabilities of these 3 cell types. The results showed that SPP1 + TAM had stronger phagocytic ability (Fig. 3E, Additional file 2: Table S2). INHBA + TAMs had stronger angiogenic ability (Fig. 3E, Additional file 2: Table S2). Macrophages fall into a “classically activated” M1/ “alternatively activated” M2 binary polarization state in vitro [37]. However, macrophages are more complex in the in vivo system, which contradicts the binary polarization classification in vitro [38]. Our results are consistent with previous studies. M1 and M2 gene signatures were coexpressed in all 5 macrophage subtypes (Fig. 3F, Additional file 2: Table S3). MACRO-INHBA, MACRO-GBP1, and MACRO-FCN1 exhibited higher M1 features (Fig. 3F). MACRO-SPP1 and MACRO-C1QC exhibited higher M2 signatures (Fig. 3F). The proportion of MACRO-SPP1 in metastatic tumors (31.82%) was significantly greater than that in primary tumors (14.44%) (Fig. 3G).

### Detection of specific signaling pathways in metastatic cancer based on the CD8 + T-cell-macrophage regulatory network

**Fig. 4:**
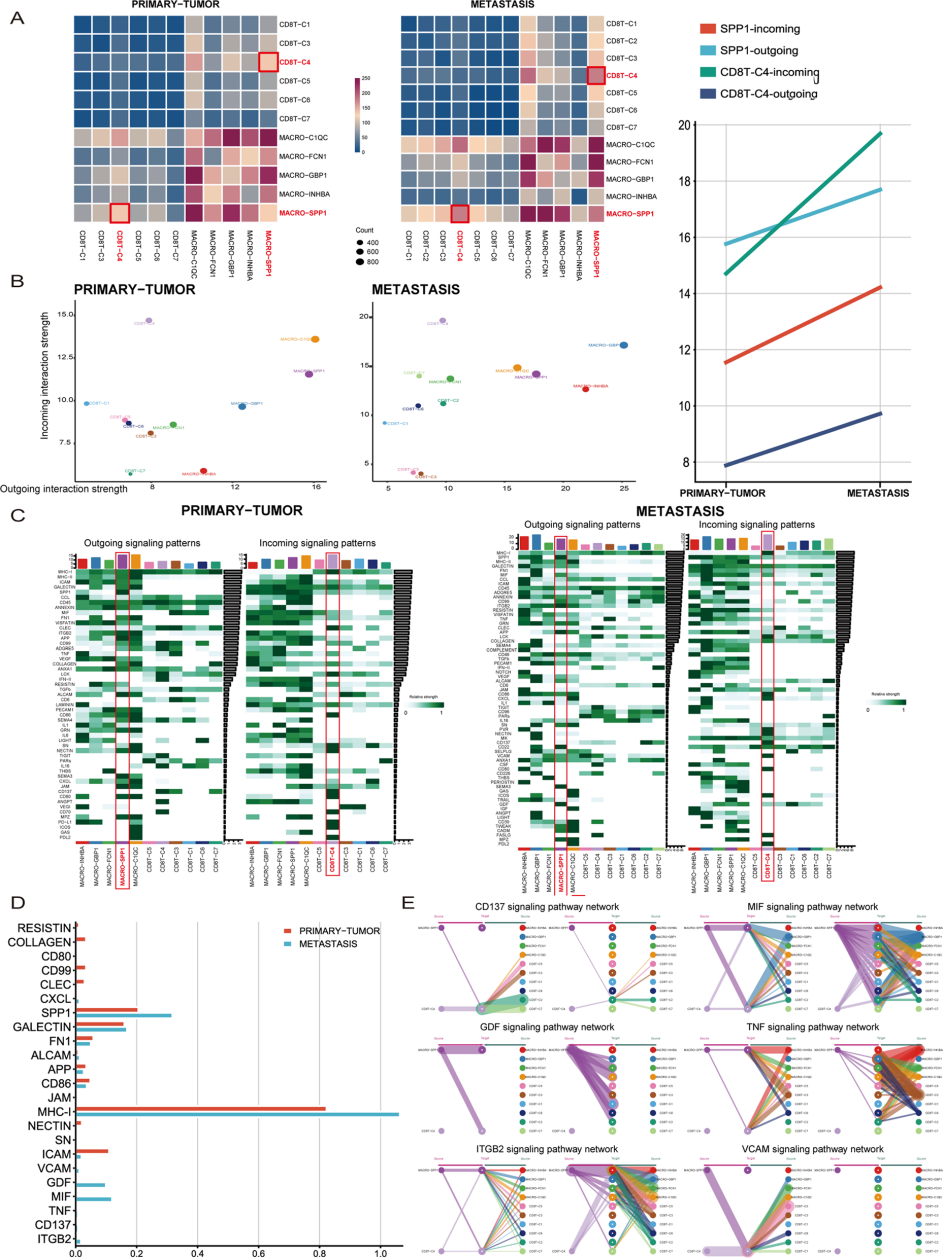
Interaction between macrophages and CD8 + T cells **A**. Heatmap showing the strength of intercellular communication in different disease states. The redder the color is, the stronger the communication between cells. The strength of the crosstalk between CD8T-C4 (exhausted CD8 + T state) and MACRO-SPP1 (SPP1 + TAMs) was significantly enhanced in the metastatic state. **B**. Incoming and outgoing communication strength of each cell subset in different disease states. The outgoing strength of MACRO-SPP1 (SPP1 + TAMs) was higher than the incoming strength. The incoming strength of CD8T-C4 (exhausted CD8 + T state) was higher than the outgoing strength. MACRO-SPP1 (SPP1 + TAMs) are more likely to be outcoming cells. CD8T-C4 cells (exhausted CD8 + T cells) are more likely to be incoming cells. **C**. Heatmap showing detailed intercellular signaling pathways between macrophages and CD8 + T cells in different states. **D**. Histograms showing signaling pathway changes from output MACRO-SPP1 (SPP1 + TAMs) to input CD8T-C4 (exhausted CD8 + T state) in different disease states. The VCAM, GDF, MIF, TNF, CD137 and ITGB2 pathways significantly appear in metastasis. **E**. Hierarchical diagram visually showing the interaction of the above signaling pathways in metastasis. The GDF signaling pathway was only specifically sent by MACRO-SPP1 (SPP1 + TAMs)

However, the mechanism of action between CD8 + T cells and tumor-associated macrophages during liver metastasis of gastric cancer remains unclear. To examine the crosstalk between macrophages and CD8 + T cells in different disease states. We first assessed the strength of cellular interactions. The results showed that the strength of the crosstalk between CD8 + exhausted T cells and SPP1 + TAMs was significantly enhanced in the metastatic state (Fig. 4A). The outgoing interaction strength of SPP1 + TAMs was significantly higher than the incoming interaction strength in metastatic carcinoma (Fig. 4B). The incoming interaction strength of CD8 + T exhausted cells was significantly higher than the outgoing interaction strength in metastatic carcinoma (Fig. 4B). We speculate that CD8 + T exhausted cells may be regulated by SPP1 + TAMs in metastatic cancer. To further understand which signaling pathways are involved in regulation, we investigated the detailed signaling pathways between macrophages and CD8 + T cells in different states (Fig. 4C). The VCAM pathway, GDF pathway, MIF pathway, TNF pathway, CD137 pathway and ITGB2 pathway specifically appeared in metastatic cancer (Fig. 4D). We visualized the interactions and networks of the above signaling pathways in metastatic cancer. The GDF signaling pathway was only specifically sent by SPP1 + TAMs (Fig. 4E).

**Fig. 5:**
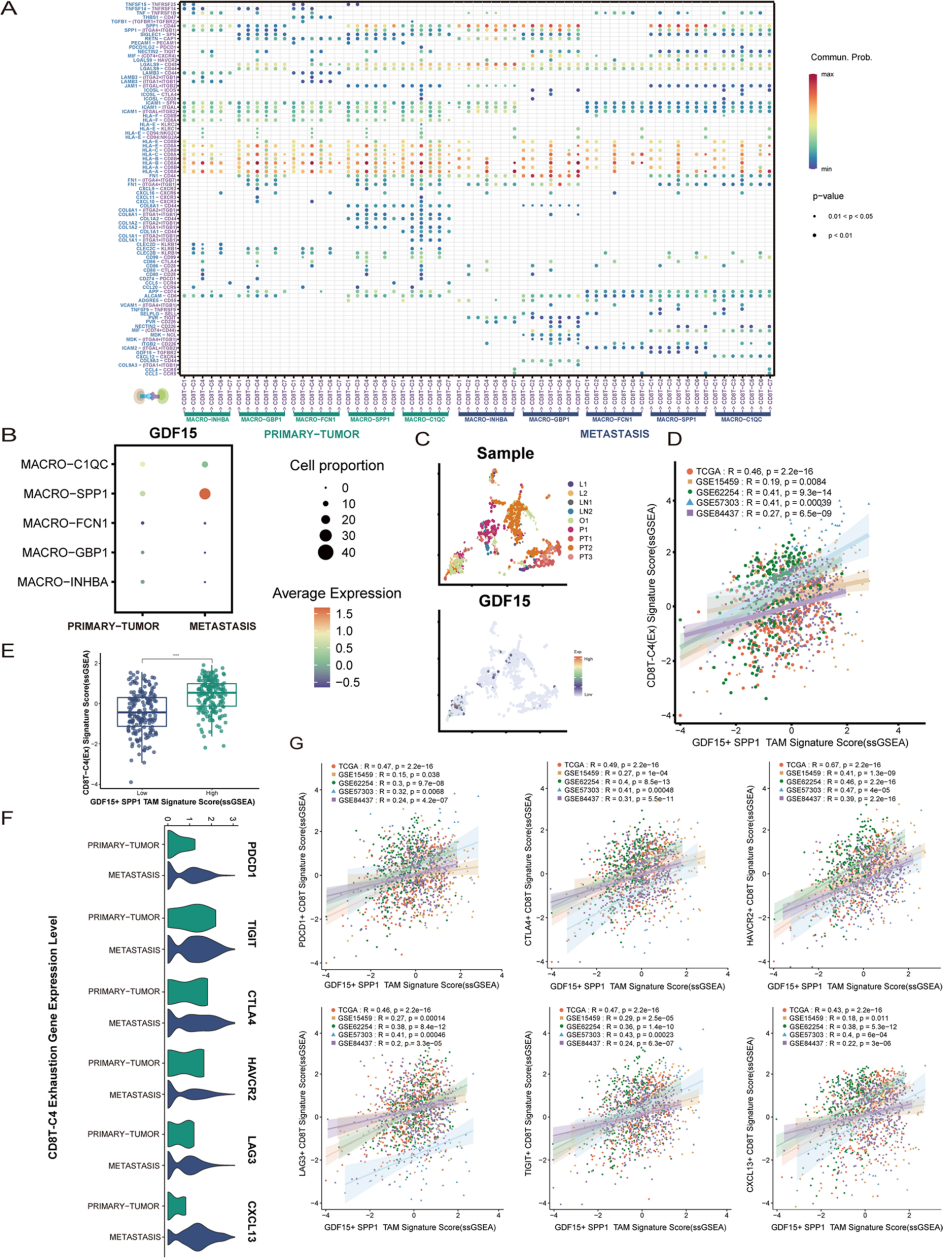
Potential ligand and receptor interactions **A**. Bubble heatmap showing ligands and receptors of cell‒cell interactions in different disease states. The GDF signaling pathway consists of GDF15-TGFBR2 (ligand receptor). **B**. Bubble heatmap showing GDF15 expression of MACRO-SPP1 (SPP1 + TAMs) in different disease states. GDF15 expression in MACRO-SPP1 (SPP1 + TAMs) is increased in metastasis. **C**. UMAP diagram showing the expression of GDF15 in different metastasis samples. GDF15 is mainly expressed in liver metastasis samples (L2). **D**. The correlation between GDF15 + SPP1 + TAM infiltration and CD8 + T exhausted cell infiltration was analyzed in the gastric cancer BULK datasets. The dotted-line plot shows a positive correlation between the GDF15 + SPP1 + TAM ssGSEA score and the CD8T-C4 (exhausted CD8 + T state) ssGSEA score. **E**. Comparison of CD8T-C4 (exhausted CD8 + T state) ssGSEA score between high- and low-expressed groups defined by the median expression level of GDF15 + SPP1 + TAM ssGSEA score in the TCGA-STAD cohort. **F**. Violin plot showing exhaustion gene expression of CD8T-C4 (exhausted CD8 + T state) in different disease states. PDCD1 and CXCL13 expression in CD8T-C4 cells (exhausted CD8 + T cells) is increased in metastasis. G. Dot-line plot showing a positive correlation between GDF15 + SPP1 + TAM ssGSEA score and exhaustion gene expression of CD8T-C4 (exhausted CD8 + T state) in the Gastric Cancer BULK Datasets. Wilcoxon test: *P < 0.05; **P < 0.01; ***P < 0.001; ****P < 0.0001

Upon further analysis of the ligand/receptor, we found that the alteration of the GDF pathway was mainly caused by the interaction of GDF15/TGFBR2 (Fig. 5A). GDF15 was highly expressed in SPP1 + TAMs in the metastatic state (Fig. 5B). GDF15 expression was highest in liver metastasis samples (L2) (Fig. 5C). GDF15 is also known as macrophage inhibitory cytokine 1 (MIC-1). First found in activated macrophages [39]. This belongs to the superfamily of transforming growth factor β (TGFβ), which is a member of the TGFβ superfamily. Previous studies have shown that GDF15 can be highly expressed in the environment of liver tumors [40]. Other studies found that GDF15 can reduce T-cell infiltration and inhibit T-cell stimulation and effector T-cell activation. Therefore, GDF15 promotes the occurrence of immune escape and tumor proliferation [41, 42]. To further analyze the inhibitory effect of GDF15 on T cells in gastric cancer, we used multigroup gastric cancer BULK datasets. The CD8 + T exhausted cell signature genes and the GDF15 + SPP1 + TAM signature genes were scored using ssGSEA (Additional file 2: Table S4). We found that the infiltration of GDF15 + SPP1 + TAMs was positively correlated with the infiltration of CD8 + exhausted T cells in all datasets (Fig. 5D). Among them, TCGA-STAD exhibited the highest correlation value (Spearman R = 0.46, p = 2.2e−16). According to the median value, we divided the TCGA-STAD GDF15 + SPP1 + TAM score into two groups. The results showed that the score of CD8 + exhausted cells was also increased under high GDF15 + SPP1 + TAM scores (Fig. 5E).

Ongoing studies indicate that CD8 + T exhausted cells exist along a developmental spectrum [43]. PD-1^Lo^ CD8 + T exhausted cells, accompanied by elevated expression of PD-1 and coinhibitory receptors, including TIM3, LAG3, TIGIT and CTLA4, continue to differentiate into loss-of-function PD-1^hi^ CD8 + T exhausted cells [44]. Once CD8 + T exhausted cells enter the PD-1^hi^ state, epigenetic enforcement prevents dedifferentiation back to the effector or PD-1^Lo^ state. Simultaneously, resistance to ICB therapy is produced, and apoptosis is eventually induced [44]. Our results show that PD-1, TIGIT, CTLA4, LAG3, and CXCL13 expression is elevated in CD8 + T exhausted cells in the metastatic state (Fig. 5F). Simultaneously, BULK verified that the GDF15 + SPP1 + TAM score exhibited a positive correlation with the score of coinhibitory receptors of CD8 + T cells (Fig. 5G, Additional file 2: Table S4). Among them, TCGA-STAD had the highest correlation value (PD-1 + CD8 + T cells: Spearman R = 0.47, p = 2.2e−16; CTLA4 + CD8 + T cells: Spearman R = 0.49, p = 2.2e−16; HAVCR2 + CD8 + T cells: Spearman R = 0.67, p = 2.2e−16; LAG3 + CD8 + T cells: Spearman R = 0.46, p = 2.2e−16; TIGIT + CD8 + T cells: Spearman R = 0.47, p = 2.2e−16; CXCL13 + CD8 + T cells: Spearman R = 0.43, p = 2.2e−16). Figure 5G, Additional file 2: Table S4).

**Fig. 6:**
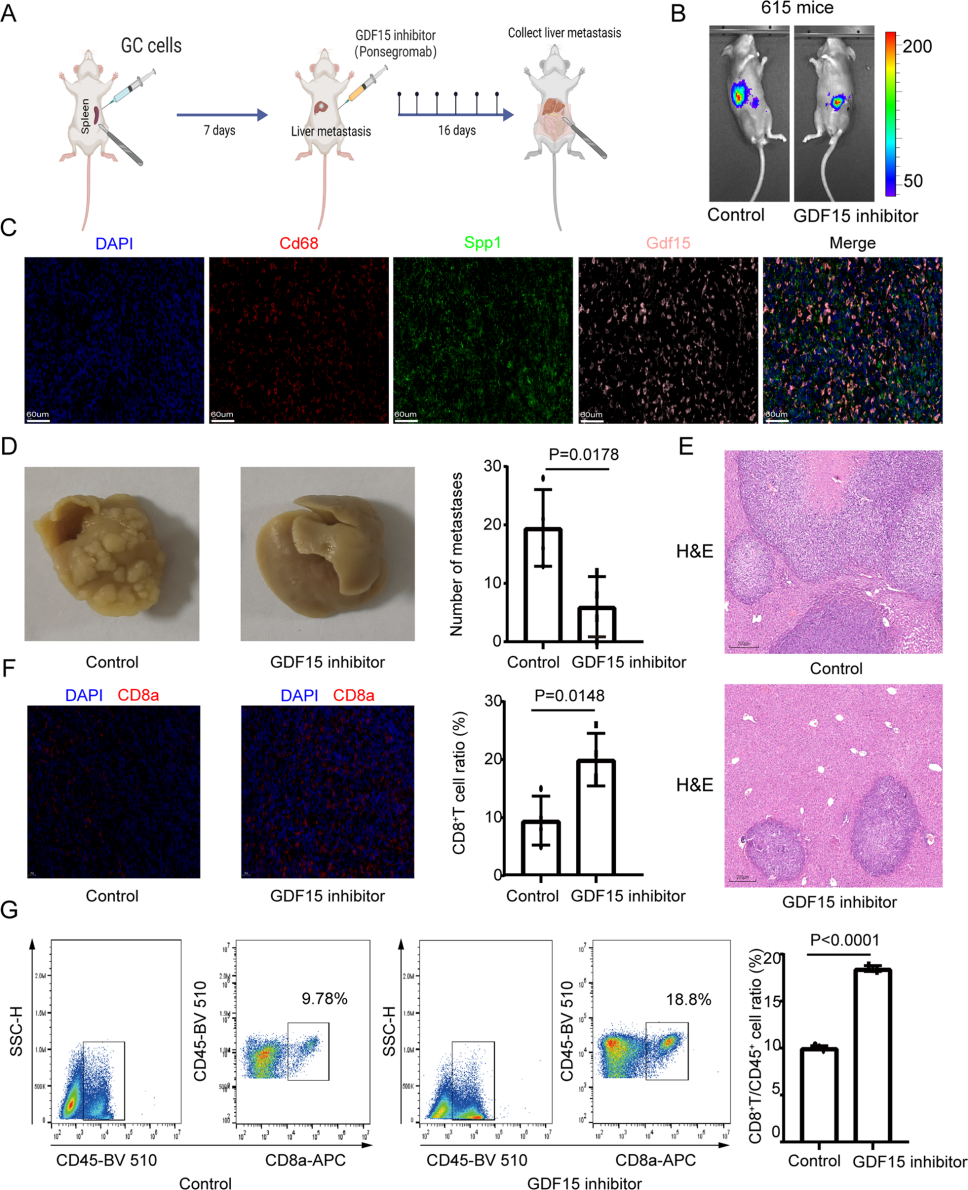
Animal Experiment Verification **A**. Schematic diagram showing the workflow of animal experiments. A total of 5 × 10^5^ (50 µl) mouse gastric cancer cells (MFCs) were injected into the subcapsule of the spleen of 8 mice (615 Mouse). Seven days after tumor development, four mice in the experimental group were intraperitoneally injected with a GDF15 inhibitor, and four mice in the control group were intraperitoneally injected with an equal volume of PBS. Thereafter, treatments were performed every 3 days for a total of 6 injections. After the last treatment, the mice were euthanized the next day, and liver metastases were collected. **B.** In vivo fluorescence imaging shows the formation of liver metastases from gastric cancer in mice. **C**. Multiplex immunofluorescence image showing the colocalization of macrophage markers (Cd68), SPP1 + TAM markers (Spp1) and Gdf15 protein. **D**. GDF15 inhibitors reduced the number of liver metastases (each group contained 4 mice). **E**. Immunohistochemical image of HE staining showing that GDF15 inhibitors can reduce the number of liver metastases. **F**. Immunofluorescence image showing that GDF15 inhibitors increase tumor-infiltrating CD8 + T cells (each group contained 4 mice). **G**. Flow cytometry showing that GDF15 inhibitors increase tumor-infiltrating CD8 + T cells (each group contained 4 mice). The number of liver metastases and flow analysis were performed by Student’s t test, and P < 0.05 was considered statistically significant

We prepared a mouse model of gastric cancer liver metastasis to further verify our above findings (Fig. 6A). Specifically, we injected gastric cancer cells under the splenic capsule. Mice were monitored weekly by fluorescence intravital imaging to observe the preparation of the liver metastasis model (Fig. 6B). After three weeks of tumor growth, the mice were sacrificed, and liver metastases were collected. Multiplex immunofluorescence results confirmed the colocalization of a macrophage marker (Cd68), an SPP1 + TAM marker (Spp1) and Gdf15 protein (Fig. 6C). Next, to further verify the immunosuppressive effect produced by GDF15, we used a GDF15 inhibitor in gastric cancer liver metastasis model mice (Fig. 6A). Specifically, we used mice as the dosing group/control group. A GDF15 inhibitor (10 mg/kg) and the same volume of PBS were injected intraperitoneally into mice. The spraying time was 2 weeks, with a total of 6 injections. The results of the study showed that GDF15 inhibitors could effectively reduce the number of liver metastases (Fig. 6B, D–E). At the same time, the results verified that the GDF15 inhibitor reversed the immunosuppressive effect and increased the infiltration of CD8 + T cells (Fig. 6F–G). These results suggest that GDF15 inhibitors may help to relieve the inhibitory effect of SPP1 + TAMs on CD8 + T cells, thereby improving the killing ability of CD8 + T cells and inhibiting liver metastasis of gastric cancer.

### CD8 + T exhausted and SPP1 + TAM interaction score generation

**Fig. 7:**
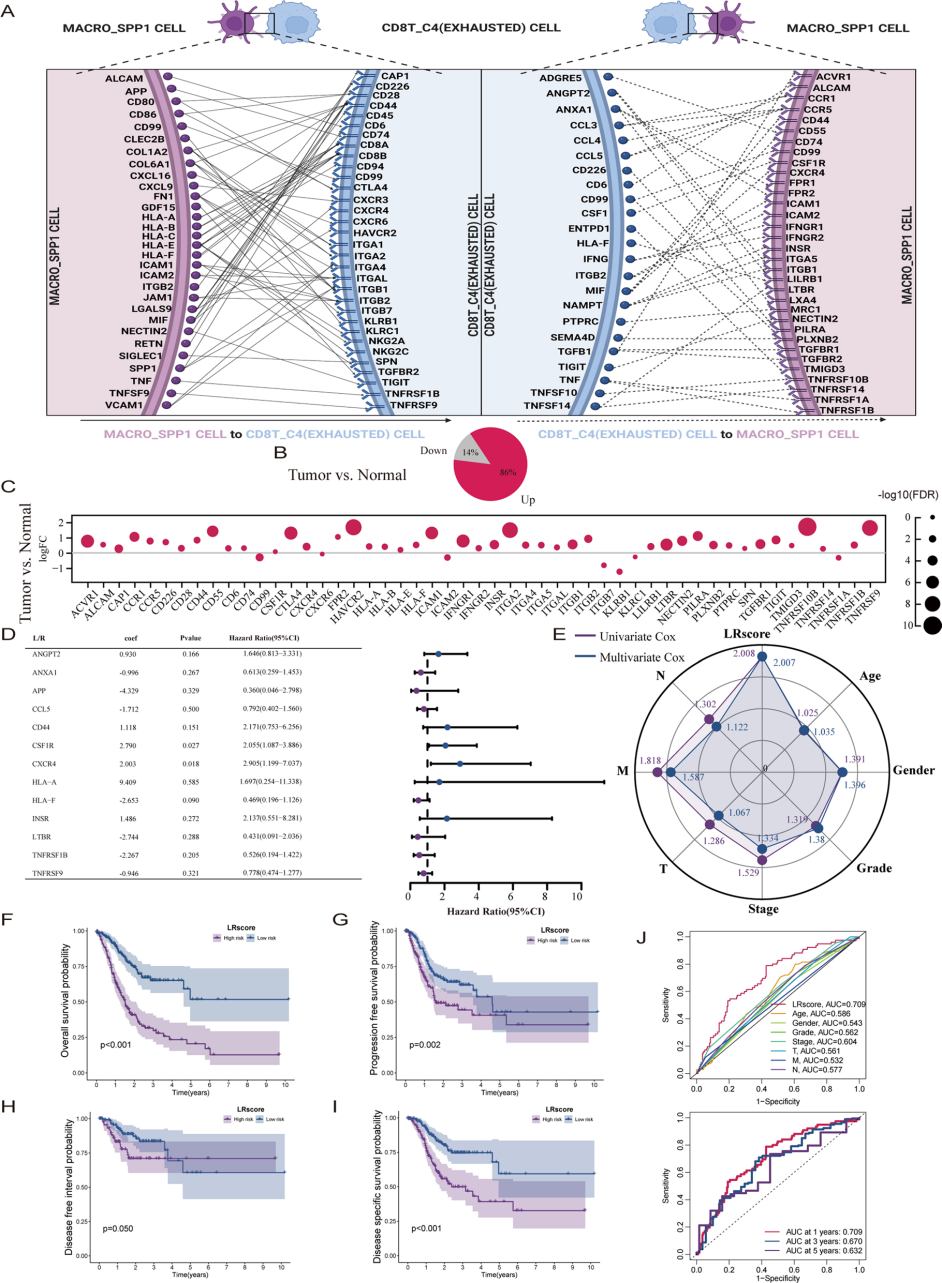
LR score for quantifying interaction. **A**. According to the results of the previous single-cell analysis. Schematic diagram showing the interaction network of ligands and receptors between MACRO-SPP1 (SPP1 + TAMs) and CD8T-C4 (exhausted CD8 + T cells). **B**. Pie chart showing ligand and receptor expression in tumor/normal tissues (TCGA-STAD) **C**. Bubble plot showing the differential expression of ligands and receptors in tumor/normal tissues (TCGA-STAD). **D**. Forest plot showing LR scores constructed based on the above ligands and receptors using LASSO and Cox regression methods (TCGA-STAD). **E**. Radar chart showing hazard ratio values based on univariate/multivariate Cox regression. The LR score had the highest multivariate and univariate HR value (TCGA-STAD). **F**–**I**. Kaplan‒Meier curve showing that the LR score is correlated with OS, PFS, DSS, and DFS (TCGA-STAD). **J**. The receiver operating characteristic (ROC) curve shows that the LR score has good evaluation performance (TCGA-STAD).

These results suggest that the intensity of the interaction between SPP1 + TAMs and CD8 + exhausted T cells in gastric cancer patients may be an important factor in evaluating the prognosis of patients. To quantify the crosstalk of CD8 + T exhausted cells with SPP1 + TAMs, we first visualized the network of SPP1 + TAMs interacting with CD8 + T exhausted cells (Fig. 7A). The levels of expression for 53 pairs of ligand/receptor (L/R) in the conduction network were measured. In the TCGA-STAD cohort, 86% of L/R were found to be upregulated in gastric cancer samples. L/R (14%) was downregulated in gastric cancer samples (Fig. 7B). Thirty-four L/R were differentially expressed between normal and gastric cancer samples (FDR < 0.05, Fig. 7C). COX regression was performed on all ligands and receptors. The LR score was built based on LASSO (Fig. 7D). The LR score had the highest multivariate and univariate HR values (Fig. 7E). Gastric cancer patients with high LR scores had worse OS (p < 0.001, Fig. 7F), PFS (p = 0.002, Fig. 7G), DFS (p = 0.05, Fig. 7H) and DSS (p < 0.001, Fig. 7I). The LR score was more effective in evaluating prognosis than the AJCC-TNM stage, pathological grade, sex, and age (Fig. 7J). The consistency of the evaluation performance at 1-, 3-, and 5 year intervals was satisfactory (Fig. 7J).

**Fig. 8:**
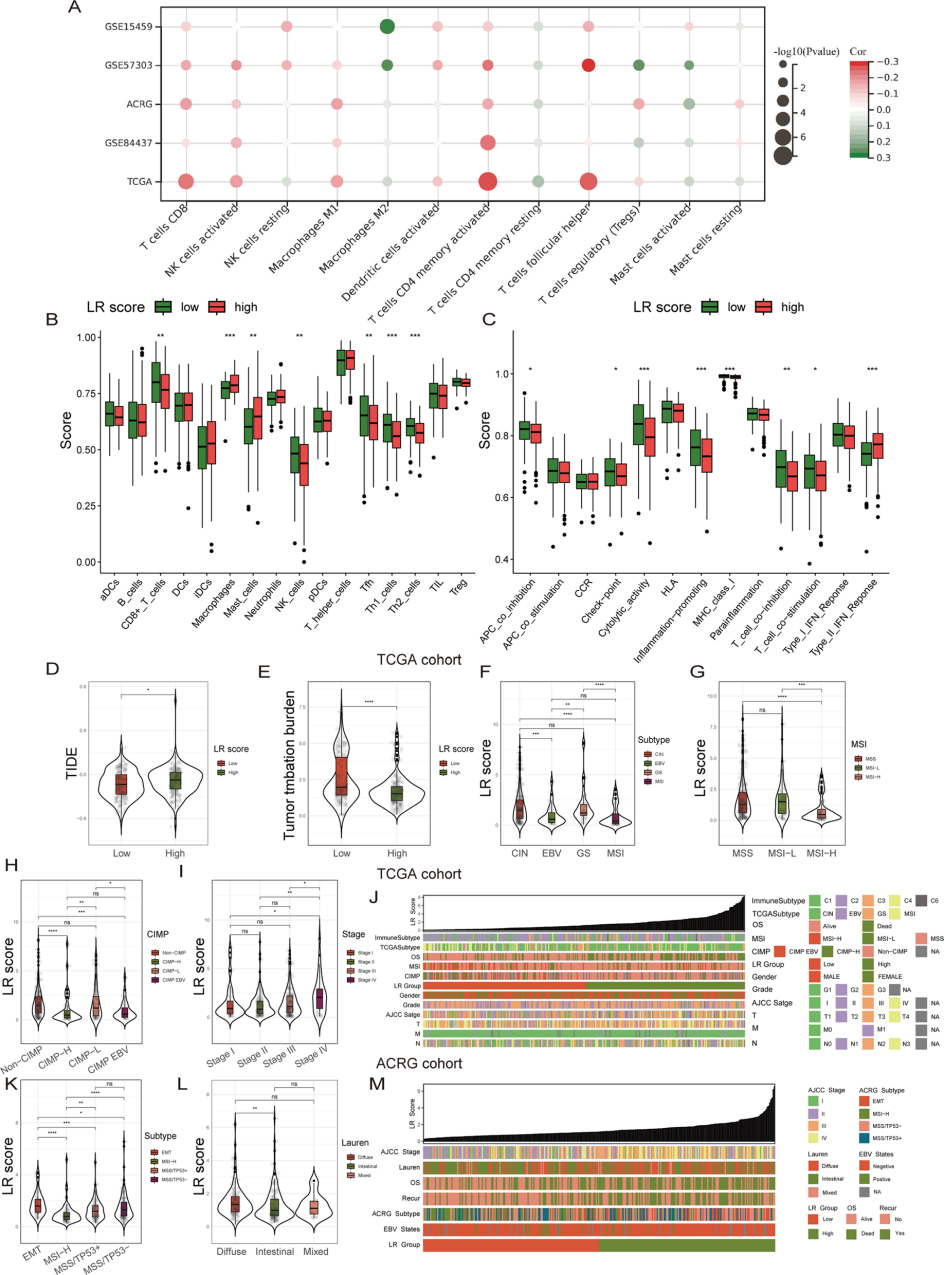
Correlation between LR score and clinical immune features. **A**. Bubble plot showing the correlation between the LR score and immune cell infiltration. The LR score was negatively correlated with most T-cell types. The LR score was positively correlated with the M2 macrophage fraction. **B**. Boxplot showing the comparison of immune cell ssGSEA scores with high/low LR score groups (TCGA-STAD). A high LR score was associated with lower CD8 + T-cell infiltration and higher macrophage infiltration. **C**. Boxplot showing the comparison of immune function ssGSEA scores with high/low LR score groups (TCGA-STAD). A low LR score had higher cell cytolytic activity and pro-inflammatory ability. **D**. Violin plot showing the comparison of TIDE scores between the high/low LR score groups. A high LR score has a higher TIDE score. High LR scores are more susceptible to immune evasion. **E**. Violin plot showing the comparison of TMB between the high/low LR score groups. Immunotherapy often yields more favorable outcomes for malignancies exhibiting elevated TMB. Low LR scores have higher TMB scores. **F**. Violin plot showing the comparison of LR scores between different TCGA subtypes. Patients with EBV and MSI may benefit from immunotherapy. GS and CIN are less likely to benefit from immunotherapy. CIN and GS had higher LR scores. Patients with EBV and MSI have lower LR scores. **G**. Violin plot showing the comparison of LR scores between different MSI subtypes. Patients with MSI-H have better immunotherapy sensitivity. MSI-H patients had lower LR scores. **H**. Violin plot showing the comparison of LR scores between different CIMP subtypes. CIMP-H and CIMP EBV had high methylation levels. High methylation levels are associated with greater immune cell infiltration. CIMP-H and CIMP-EBV had lower LR scores. **I**. Violin plot showing the comparison of LR scores between different TNM stages. Individuals with an advanced clinical stage exhibited an elevated LR score. **J**. The clinical heatmap shows the distribution of the LR score and various molecular subtypes in the TCGA cohort. **K**. Violin plot showing the comparison of LR scores between different ACRG subtypes. The prognosis for the MSI-H type is the most favorable, while the prognosis for the EMT type is the worst favorable. MSI-H exhibits the lowest LR score. The EMT type had the highest LR score. **L**. Violin plot showing the comparison of LR scores between different Lauren types. The diffuse type has the worst prognosis and is more prone to distant metastasis. Individuals diagnosed with diffuse gastric cancer exhibited the most elevated LR score. **M**. Clinical heatmap showing the distribution of the LR score and various molecular subtypes in the ACRG cohort. Wilcoxon test: *P < 0.05; **P < 0.01; ***P < 0.001; ****P < 0.0001

Afterward, we investigated the correlation of the LR score with immune characteristics as well as clinical molecular subtypes. The LR score was negatively correlated with most T-cell types (Fig. 8A). The LR score was positively correlated with the M2 macrophage fraction (Fig. 8A). In addition, a high LR score was associated with lower CD8 + T-cell infiltration and higher macrophage infiltration (Fig. 8B). A low LR score had higher cell cytolytic activity and pro-inflammatory ability (Fig. 8C). These results are consistent with the results of the single-cell analysis. More importantly, a high LR score was associated with a higher TIDE score (Fig. 8D). It is evident that gastric cancer patients who have high LR scores are more susceptible to immune evasion. Furthermore, individuals with a low LR score in gastric cancer might exhibit heightened responsiveness to ICB (Fig. 8D). TMB is considered to predict the efficacy of ICB drugs and functions as a biomarker in a variety of tumors. Immunotherapy often yields more favorable outcomes for malignancies exhibiting elevated TMB [45]. Our results show that patients with a low LR score have a higher TMB score (Fig. 8E). The National Cancer Institute released the TCGA Subtypes for Gastric Cancer in 2014 [34]. Gastric cancer is divided into four types: MSI (microsatellite instability), GS (genome stable), EBV (Epstein‒Barr virus infection) and CIN (chromosomal instability). Among these types, EBV and MSI may benefit from immunotherapy. However, patients with GS and CIN are less likely to benefit from immunotherapy. Our results show that CIN and GS have higher LR scores (Fig. 8F). However, EBV and MSI had lower LR scores (Fig. 8F). Multiple research studies have validated that the presence of microsatellite instability in gastric cancer can serve as a reliable indicator for determining the effectiveness of immunotherapy [46, 47]. Patients with MSI-H have better immunotherapy sensitivity. Our results showed that MSI-H patients had lower LR scores (Fig. 8G). The CIMP (CpG Island Methylator Phenotype) is based on subtypes of gastric cancer CpG island methylation levels [48, 49]. Non-CIMP and CIMP-L have low methylation levels. However, CIMP-H and CIMP EBV had high methylation levels. High methylation levels are associated with greater immune cell infiltration [50]. Our results showed that CIMP-H and CIMP-EBV had lower LR scores (Fig. 8H). The above results all indicated that LR scores were highly correlated with immune features. To some degree, the interaction score can help predict the effectiveness of immunotherapy.

Furthermore, individuals with an advanced clinical stage exhibited an elevated LR score (Fig. 8I). Additionally, this result validated the prognostic significance of the LR score in patients. The figure illustrates the varying LR score distributions and clinical molecular subtype characteristics among TCGA-STAD patients (Fig. 8J). To circumvent the constraints of a solitary database. Next, we conducted the analysis on the dataset from the Asian Cancer Research Group (ACRG, GSE62254). In 2015, the ACRG molecular subtype classification was published by the Asian Cancer Research Organization [51]. There are four categories of gastric cancer: MSI-H (microsatellite unstable), EMT (epithelial-mesenchymal transition), MSS (microsatellite stable)/TP53- and MSS (microsatellite stable)/TP53 + . The prognosis for the MSI-H type is the most favorable, while the prognosis for the EMT type is the worst favorable. According to our findings, MSI-H exhibits the lowest LR score (Fig. 8K). The EMT type had the highest LR score (Fig. 8K). The classification by Lauren categorizes gastric cancer into three types: diffuse, intestinal, and mixed. The diffuse type has the worst prognosis and is more prone to distant metastasis. According to our findings, individuals diagnosed with diffuse gastric cancer exhibited the most elevated LR score (Fig. 8L). The aforementioned findings once again demonstrated a strong correlation between interaction scores and clinical molecular subtypes. This illustrates the varying LR score distributions and clinical molecular subtype characteristics among ACRG patients (Fig. 8M).

## Discussion

ICB drugs relieve the inhibitory effect of T cells in tumor tissues and reactivate T cells to kill tumor cells, bringing new benefits to patients with advanced gastric cancer who are undergoing immunotherapy [11–15]. Nevertheless, only a limited proportion of individuals diagnosed with gastric cancer experience the benefits of immunotherapy [16]. Recently, it was found that liver metastasis of gastric cancer can significantly reduce the efficacy of immunotherapy in patients [19–21]. The high degree of heterogeneity of tumors and the surrounding complex ecosystem are responsible [17]. Therefore, there is an urgent need to explore the immune status in the tumor microenvironment of patients with liver metastases and reveal the deeper mechanisms affecting CD8 + T cells.

Several recent studies found that TAMs significantly affect tumor metastasis and found more TAMs in liver metastases [19]. However, the mechanism of TAM interaction with CD8 + T cells during the occurrence and progression of gastric cancer liver metastasis remains unclear. To elucidate this mechanism and understand the microenvironment and cell‒cell interactions in different disease states, our study generated a comprehensive single-cell landscape of healthy stomach, gastric primary cancer, and metastatic cancer. We found that the proportion of SPP1 + TAMs increased in metastasis by reclustering TAMs. Relevant research reports have shown that SPP1 + TAMs are thought to have immunosuppressive properties [25]. However, the involvement of SPP1 + TAMs in gastric cancer metastases remains unclear.

Our study generated a landscape of healthy stomach, primary gastric cancer, and metastatic cancer. These results are used to understand the microenvironment and cell‒cell interactions in different disease states. Initially, our attention was directed toward variations in macrophage subsets and alterations in CD8 + T-cell subsets in different disease states. In metastasis, there was an observed increase in the proportions of exhausted CD8 + T cells and SPP1 + TAMs. Concurrently, there was a notable increase in the level of interaction between exhausted CD8 + T cells and SPP1 + TAMs during the process of metastasis. These findings prompted us to pay further attention to the differences. SPP1 + TAMs showed higher output strength in metastasis. CD8 + T exhausted cells showed higher input strength in metastasis. CD8 + T exhausted cells in metastatic cancer may be regulated by SPP1 + TAMs. Interestingly, the GDF signaling pathway is highly activated in the metastatic state. It is specifically emitted by SPP1 + TAMs. GDF15-TGFBR2 is the ligand/receptor for GDF pathway changes. GDF15 is highly expressed in SPP1 + TAMs in liver metastasis samples. Previous studies have shown that GDF15 can be highly expressed in the environment of liver tumors [40]. GDF15 can reduce T-cell infiltration and inhibit T-cell stimulation and effector T-cell activation. GDF15 promotes immune escape and tumor proliferation [41, 42]. The aforementioned findings indicate that GDF15 might play a crucial role in suppressing the immune microenvironment of liver metastases.

To further explore whether GDF15 affects the immune status of gastric cancer. We further analyzed the inhibitory effect of GDF15 on T cells in gastric cancer using multiple gastric cancer BULK datasets. We discovered a positive correlation between the infiltration of GDF15 + SPP1 + TAMs and the infiltration of exhausted CD8 + T cells. High expression of GDF15 can enhance the expression of inhibitory receptors in exhausted CD8 + T cells and finally induce the apoptosis of exhausted cells [43, 44]. To validate our above analysis, we used a gastric cancer liver metastasis model for validation. In the liver metastasis model, we successfully observed GDF15 + SPP1 + TAMs and significantly inhibited the liver metastasis ability of gastric cancer after using GDF15 blockade. We then further confirmed through immunofluorescence and flow cytometry analysis that inhibiting GDF15 significantly increased the infiltration level of CD8 + T cells in liver metastases and successfully reversed the immunosuppressive effect. The above results suggest that inhibiting the GDF15 protein secreted by SPP1 + TAMs may be an important way to improve the efficacy of immunotherapy and improve liver metastasis in patients with gastric cancer.

Due to the importance of CD8 + T exhausted cell interactions with SPP1 + TAMs, we generated a score to quantify the crosstalk between SPP1 + TAMs and exhausted CD8 + T cells. Our results further confirm that a high LR score will lead to a worse prognosis. In addition, individuals exhibiting a high LR score demonstrated a reduced presence of CD8 + T cells and an increased presence of M2 macrophages. These results are consistent with the results of the single-cell analysis. Furthermore, we explored the correlation of this quantitative score with clinical molecular subtypes. The LR score showed a strong correlation with the existing mainstream TCGA subtypes, ACRG subtypes, and MSI subtypes. The LR score may also guide the prediction of prognosis and efficacy of immunotherapy.

There is no denying that our research has certain limitations. First, our research relies on public single-cell datasets. It lacks a sufficient number of single-cell samples. This would indicate bias in our results. Second, it is very difficult to obtain clinical samples of liver metastases from patients with gastric cancer. We could not directly, experimentally verify that GDF15 + SPP1 + TAMs are highly activated in liver metastases. Finally, our study only characterizes the communication potential between SPP1 + TAMs and CD8 + T exhausted cells. However, why this crosstalk is highly activated in the metastatic state and the specific mechanism of the immunosuppressive effect remain unclear. This requires us to conduct further in-depth research.

## Conclusions

To summarize, our findings indicate that the crosstalk between SPP1 + TAMs and CD8 + exhausted T cells has a significant immunosuppressive impact within the microenvironment of gastric metastases. Through GDF15-TGFBR2, SPP1 + TAMs have the ability to enhance the expression of coinhibitory receptors, leading to apoptosis in exhausted CD8 + T cells. Drugs that block GDF15 aid in reversing the immunosuppressive microenvironment. The prognosis and effectiveness of immunotherapy can be predicted using a quantified interaction score.

### Supplementary Information

**Additional file 1: Figure S1.** Single-cell dataset quality control and annotation. **A**. Violin plot showing three quality parameters of the dataset. **B**. Bubble heatmap showing the expression of classic cell markers in different cell types

**Additional file 2: Table S1.** Benign/Malignant Epithelial Functional Signature Genes. **Table S2.** Phagocytosis/Angiogenesis Signature Genes. **Table S3.** M1/M2 Signature Genes. **Table S4.** Specific Cell Type Signature Genes.

## Data Availability

The original data presented in the study are included in the article/Supplementary Materials, and further inquiries can be directed to the corresponding authors.

## Abbreviations

ACRG: Asian Cancer Research Group
AJCC: American Joint Committee on Cancer
CIN: Chromosomal instability
CIMP: CpG Island methylator phenotype
CNV: Copy number variation
DFS: Disease-free survival
DSS: Disease-specific survival
EBV: Epstein‒Barr virus infection
EDTA: Ethylenediamine tetraacetic acid
EMT: Epithelial-mesenchymal transition
FBS: Fetal bovine serum
FDR: False discovery rate
GDF15: Growth differentiation factor 15
GEO: Gene expression omnibus
GS: Genome stable
HR: Hazard ratio
ICB: Immune checkpoint blockade
LASSO: Least absolute shrinkage and selection operator
L/R: Ligand/Receptor
MIC-1: Macrophage inhibitory cytokine 1
MSI: Microsatellite instability
MSS: Microsatellite stable
OS: Overall survival
PBS: Phosphate-buffered saline
PD-1: Programmed cell death protein-1
PD-L1: Programmed cell death 1 ligand-1
PFS: Progression-free survival
SPP1: Secreted phosphoprotein 1
ssGSEA: Single sample gene set enrichment analysis
TAMs: Tumor-associated macrophages
TCGA-STAD: The cancer genome atlas—stomach adenocarcinoma
TIDE: Tumor immune dysfunction and exclusion
TMB: Tumor mutational burden
TNM: Tumor node metastasis
TGFβ: Transforming growth factor β
UMAP: Uniform manifold approximation and projection
