# Supplementary figures and images for: Potential crosstalk between SPP1 + TAMs and CD8 + exhausted T cells promotes an immunosuppressive environment in gastric metastatic cancer
Source: J Transl Med. 2024 Feb 16;22:158. doi: 10.1186/s12967-023-04688-1 (PMC10870525; doi:10.1186/s12967-023-04688-1)

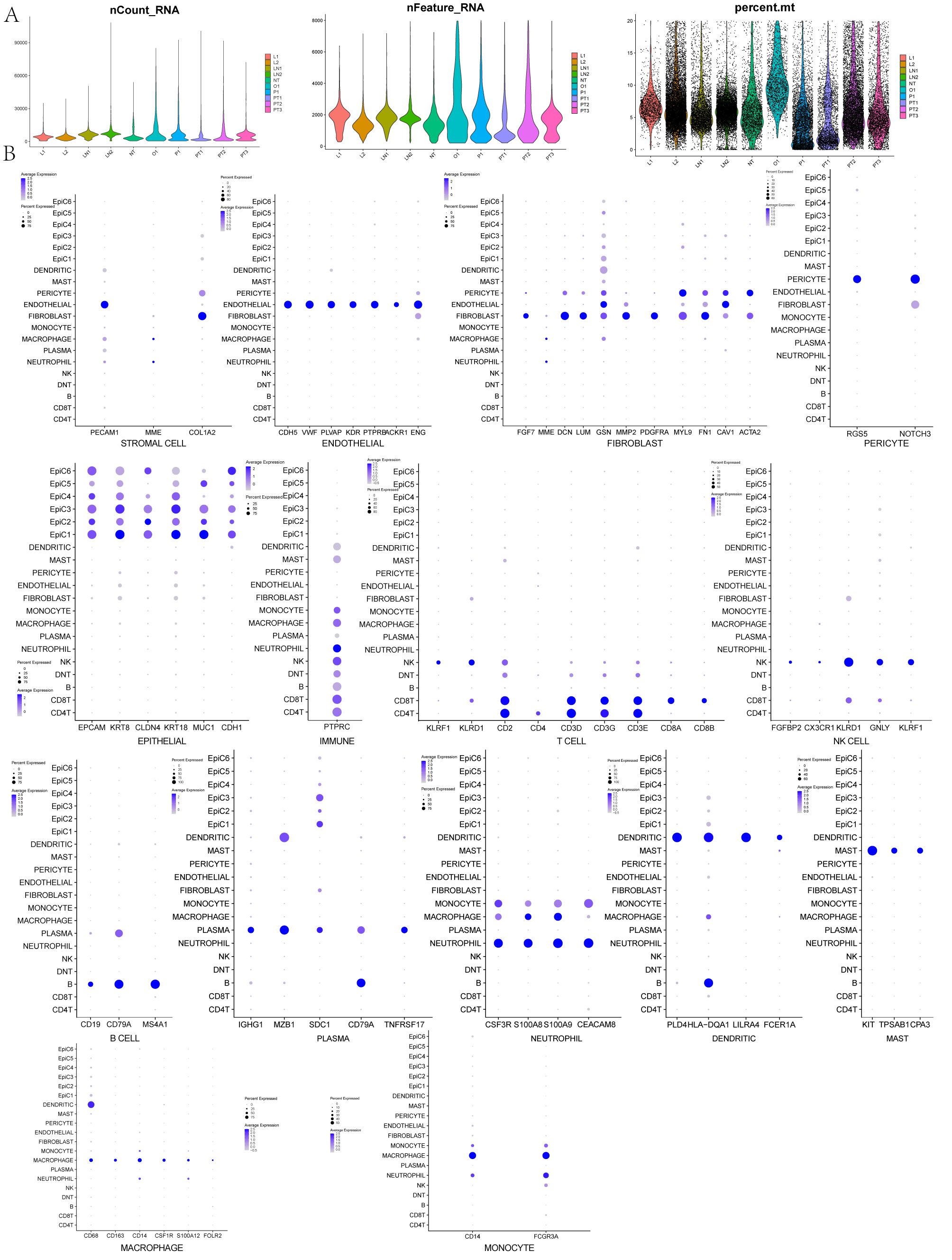

Supplement: Supplementary file 1 — Additional file 1: Figure S1. Single-cell dataset quality control and annotation. A. Violin plot showing three quality parameters of the dataset. B. Bubble heatmap showing the expression of classic cell markers in different cell types [file 12967_2023_4688_MOESM1_ESM.tif]
